# The Impact of Adjuvanted Influenza Vaccine on Disease Severity in the US: A Stochastic Model

**DOI:** 10.3390/vaccines11101525

**Published:** 2023-09-26

**Authors:** Stephen I. Pelton, Joaquin F. Mould-Quevedo, Van Hung Nguyen

**Affiliations:** 1 Chobanian and Avedesian School of Medicine, Boston University, Boston, MA 02118, USA; 2 Seqirus USA Inc., Summit, NJ 07901, USA; 3 VHN Consulting Inc., Montreal, QC H2V 3L8, Canada

**Keywords:** influenza vaccine, aQIV, US, hospitalization, mortality, severity

## Abstract

Influenza can exacerbate underlying medical conditions. In this study, we modelled the potential impact of an egg-based quadrivalent influenza vaccine (QIVe) or adjuvanted QIV (aQIV) on hospitalizations and mortality from influenza-related cardiovascular disease (CVD), respiratory, and other complications in adults ≥65 years of age in the US with underlying chronic conditions. We used a stochastic decision-tree model, with 1000 simulations varying input across predicted ranges. Due to the variable nature of influenza across seasons and differences in published estimates for input parameters, data are presented as 95% confidence intervals. Compared with no vaccination, use of aQIV would prevent 135,450–564,360 hospitalizations and 1612–29,226 deaths across outcomes evaluated. Overall, aQIV prevented 1071–18,388 more hospitalizations and 85–1944 more deaths than QIVe. By routine seasonal vaccination against influenza, a substantial number of severe influenza-associated complications and deaths, caused by direct influenza symptoms or by exacerbation of chronic conditions, can be prevented in high-risk adults ≥65 years of age in the US.

## 1. Introduction

Despite the public health advocacy for universal influenza vaccination, influenza vaccine uptake varies between 37% in the 18 to 49 age cohort to 73% in individuals greater than 65 years of age [1]. The disease burden between 2010 and 2020 is estimated to have caused 140,000–710,000 hospitalizations and 12,000–52,000 deaths annually in the United States, suggesting higher vaccine uptake and new formulation are needed in at-risk populations [2]. Although influenza activity was minimal during the 2021–2022 season, preliminary estimates for the 2022–2023 season indicate a disease burden comparable to pre-pandemic levels, with an estimated 290,000–620,000 hospitalizations and 18,000–54,000 deaths through February 2023 [3].

Influenza disproportionately affects older adults, in part related to immunosenescence, the age-related decline in immune system function, as well as the relatively large proportion with comorbidities [4,5]. In the past two pre-pandemic seasons (2018–2019 and 2019–2020), despite low rates of symptomatic illness and fewer general practitioner visits compared with other age groups, 44–54% of hospitalizations and 63–77% of deaths from influenza in the US were in adults ≥65 years of age [6,7]. In the current season (2022–2023), influenza hospitalization rates in adults ≥65 years of age have been estimated at 178.6 per 100,000, with rates of 326 per 100,000 in those ≥85 years of age [8]. In addition to direct morbidity and mortality from symptomatic disease, influenza infection has also been associated with exacerbations of underlying chronic medical conditions, as well as increased risk of neurological, cardiovascular, and respiratory complications including pneumonia, acute myocardial infarction, and stroke [9,10,11,12]. Previous studies have shown that seasonal influenza vaccination can reduce the risk of these complications, particularly in patients with underlying comorbidities [13,14,15,16].

In contrast to previous seasons, adjuvanted and high-dose vaccines were preferentially recommended in 2022–2023 for adults ≥65 years of age [17]. The addition of adjuvants to vaccines can help to enhance the immune response to vaccination, increasing effectiveness. aQIV, an adjuvanted quadrivalent influenza vaccine (QIV), contains MF59^®^ adjuvant, which increases vaccine effectiveness by recruiting immune cells, promoting IgG isotype-switching, and inducting inflammatory cytokines [18]. While there have been no head-to-head comparisons performed evaluating the effectiveness of aQIV vs. standard dose QIVs, aQIV has demonstrated greater effectiveness in preventing influenza and related outcomes than non-adjuvanted QIVs in clinical trials and real-world evaluations [19,20,21]. While breakthrough infections occur in patients who have been vaccinated, vaccination can modify disease severity. A 26% reduction in the risk of ICU admission in adults who have been hospitalized from influenza and a 31% reduction in the risk of death have been reported [22]. Similarly, in a retrospective cohort study of the relative vaccine effectiveness (rVE) of aQIV and adjuvanted trivalent influenza vaccines (aTIV) during the 2019–2020 season, the adjuvanted vaccines were associated with lower rates of inpatient and outpatient influenza, with an rVE of 27.8% versus non-adjuvanted standard dose vaccines and 14.3% versus high-dose vaccines in adults ≥65 years [23]. While the adjuvanted vaccines and high-dose formulations were comparable at preventing hospitalization of older adults from influenza, the rVE versus standard-dose vaccines was 13.4%, indicating a clear benefit in reducing influenza severity. Similarly, other studies which evaluated aTIV in older adults in Europe have also demonstrated reduced risk of hospitalization from influenza as well as associated complications including cerebral and cardiovascular events and pneumonia [24,25,26].

The objective of the current analysis was to estimate the reduction in influenza severity following immunization. We modelled the potential impact of aQIV or a non-adjuvanted egg-based QIV (QIVe) on hospitalizations and mortality from influenza-related cardiovascular, respiratory, and other complications in adults ≥65 years of age in the US. We used a stochastic approach to estimate the range of impacts on each outcome compared with no influenza vaccine and with QIVe in adults ≥65 years, based on pre-COVID-19 pandemic seasonal influenza surveillance data from the US.

## 2. Methods

### 2.1. Model Input Parameters

**Figure 1:**
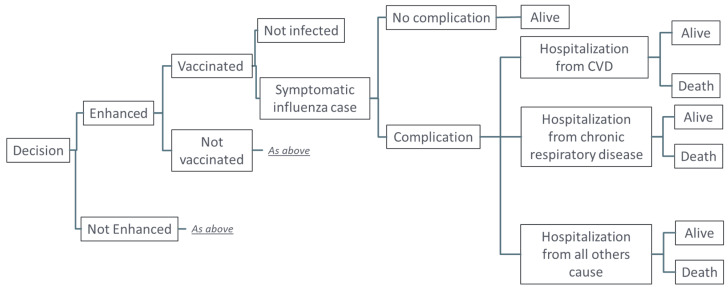
Model structure.

As literature sources vary substantial in parameter estimates (see discussion), in this analysis we used a stochastic decision tree model, based on the model by Mangen et al. 2015 [27], as outline in Figure 1. Three key outcomes were evaluated within one influenza season for vaccinated or unvaccinated patients ≥65 years of age with symptomatic influenza who developed complications (exacerbations of underlying diseases): hospitalization from underlying cardiovascular disease (CVD); hospitalization from chronic respiratory disease; and hospitalization from all other causes. Other age groups were not considered in this analysis or included in the model. The model was calibrated based on published data on the burden of influenza in adults ≥65 years of age over recent influenza seasons, excluding those during the COVID-19 pandemic. To reflect the variability in influenza attack rate in the age cohort across seasons, we have used a range of 4–10% in our stochastic approach [28]. Prior to calibration, the probability of no complications from symptomatic influenza infection was set at 76%, based on data from Molinari et al. 2007 [29], which was adjusted to 83.7% post-calibration, based on the published data from the CDC in this age group [28]. Probability of hospitalization from CVD and chronic respiratory diseases were assumed to range from 17% to 50% and from 9% to 26%, respectively [30]. The probability of death following hospitalization was 5% to 14% for all outcomes, based on data from Mauskopf et al. 2013 [31]. Estimates of vaccine effectiveness (VE) against hospitalization were based on data from the 2019–2020 season in the US, as data were not available for the 2017–2018 season [32]. Relative vaccine effectiveness (rVE) of aQIV versus QIVe on CVD and respiratory outcomes was 13%, based on data on all-cause outcomes from the 2019–2020 season [23].

### 2.2. Stochastic Simulation

As there was substantial variability in parameter estimates across literature sources, the analysis was performed using a stochastic simulation, varying within a range for each parameter following a predefined distribution (Table 1). Each parameter was simulated independently from the other parameters, with no correlations among parameters assumed. A total of 1000 simulations were run, with results presented as 95% confidence intervals of median values across simulations.

### 2.3. Outcomes Evaluated

Rates of hospitalization and death due to CVD, respiratory disease, and other complications of influenza were estimated for a “no vaccination” scenario compared with vaccination with either QIVe or aQIV. Vaccine coverage for both the QIVe and aQIV scenarios was assumed to be 65%, based on estimates of the coverage in the US prior to the COVID-19 pandemic [33]. All outcomes were evaluated descriptively with no formal hypotheses tested. Influenza burden was based on a high incidence season to maximize the power to describe differences in hospitalization and mortality outcomes between the no-vaccine and vaccine scenarios.

### 2.4. Software

The model was developed using R 4.2.1 software and C++, as outlined in Nguyen et al. 2022 [34], predominantly using the following packages and corresponding libraries: Rcpp 1.0.9, RcppArmadillo 0.11.2.3.1, and RcppGSL 0.3.11.

## 3. Results

In the scenario where there was no vaccination of adults ≥65 years, our model estimates that 44,347 to 196,134 individuals in this age group would be hospitalized for respiratory complications of influenza (median: 98,794); 82,306 to 378,236 would be hospitalized for CVD complications (median 190,829), and 135,789 to 533,813 for other complications (median 299,343) (Table S1). No vaccination of adults ≥65 years was also estimated to result in 3139 to 19,996 deaths from respiratory illnesses (median: 9303), 6379 to 40,583 deaths from CVD (median: 17,349) and 10,051 to 56,806 deaths from other complications (median: 26,235). For cases of hospitalized influenza, the case fatality rate was 9% among unvaccinated individuals.

**Figure 2:**
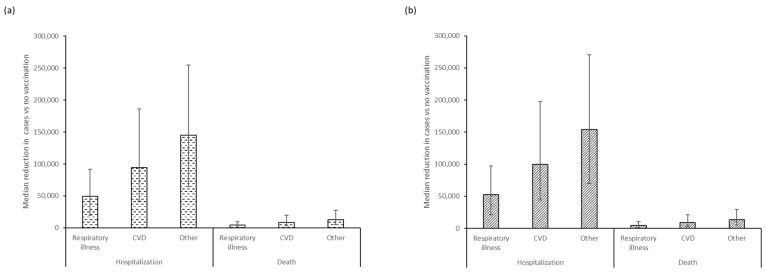
Median (95% confidence interval) reductions in hospitalized cases and deaths from respiratory illnesses, cardiovascular disease, and other causes prevented by (**a**) QIVe and (**b**) aQIV vaccination, compared with the no-vaccination scenario in adults ≥65 years of age.

The use of either QIVe or aQIV in adults ≥65 years substantially reduced the estimated incidence of hospitalization and deaths due to influenza complications (Table S1). Compared with no vaccination, use of QIVe would result in a reduction of 19,584 to 254,794 hospitalizations, across the outcomes analyzed, and 1517 to 27,548 deaths (Figure 2a). The use of aQIV would lead to a reduction of 21,354 to 270,151 hospitalizations and 1612 to 29,226 deaths (Figure 2b). Incremental differences between the two vaccines estimated further reductions in hospitalizations from aQIV vs. QIVe of 1071 to 18,388 and of 85 to 1944 deaths across the three evaluated outcomes (Table S2). Median influenza deaths fell nearly 50% from 53,000 to 29,500 but case fatality rates remained unchanged.

## 4. Discussion

While influenza is primarily a respiratory disease, it can cause broader consequences such as cardiovascular events, and exacerbations of underlying conditions such as chronic kidney disease, neurological disorders, and diabetes, resulting in excess hospitalizations and mortality. Increased risk of hospitalization leads to greater risk of disability [35], reduced quality of life [9], and increased mortality rate [36] following hospital discharge. Based on the estimated ranges generated in this model, use of QIVe or aQIV substantially reduces the number of hospitalized cases and deaths due to respiratory, cardiovascular, and other complications of influenza in adults ≥65 years of age compared with the no-vaccination scenario of this age group.

Historically, vaccine research has primarily focused on disease prevention, measured as the effectiveness in decreasing cases of disease [37]. The COVID-19 pandemic has brought considerable attention to the mechanisms by which vaccination elicits disease attenuation. Many vaccinated patients experience breakthrough symptomatic SARS-CoV-2 infections but these tended to be mild, with rates of all-cause hospitalization and ICU admissions considerably lower in vaccinated compared with non-vaccinated individuals, indicating a protective role in reducing disease severity and complications rather than preventing infection [38]. Recent studies evaluating the impact of vaccination on influenza disease severity have used hospital and ICU admissions as a proxy for disease severity and have found substantially reduced risk of these outcomes in vaccinated vs. non-vaccinated individuals. In a meta-analysis of studies evaluating disease severity by vaccination status, influenza vaccination was associated with a 26% reduction in odds of ICU admission in adults with influenza-associated hospitalization, and a 31% reduced risk of death [22]. However, vaccination was not significantly associated with a decreased risk of overall hospitalization in patients initially managed as outpatients, or in reducing the risk of pneumonia among hospitalized patients with influenza. Longitudinal analysis of six influenza seasons in Spain has also demonstrated similar impacts on lowering ICU admission and death rates from influenza by approximately 23%, even when vaccination did not prevent infection or symptomatic disease requiring hospitalization [39]. Similarly, in a separate study in New Zealand, influenza vaccination was associated with a 59% reduction in the risk of ICU admission and with shorter ICU length of stays, but not with hospital length of stay or risk of pneumonia events [40]. A recent study comparing high-dose and standard-dose vaccines in older adults in Denmark has demonstrated an incremental benefit of the high-dose formulation in older adults, with a 49% reduction in mortality risk and 64% reduction in influenza/pneumonia hospitalization compared with standard-dose vaccines [41]. How vaccination against influenza provokes disease attenuation is not yet completely understood, but the mechanisms are probably multifactorial potentially including reducing viral replication, faster clearance of virally-infected cells, and slowing the onset of illness, allowing sufficient time for activation of anamnestic immune responses [42].

Older adults and those with pre-existing comorbidities are at increased risk of experiencing severe complications of influenza infection [43]. Multiple mechanisms could explain an increased cardiovascular risk after influenza infection, including atherosclerotic plaque destabilization and subsequent thrombosis, deposition of immune complexes in atherosclerotic plaques, and elevation of macrophage circulation into the arteries resulting in coronary vascular events. Acute heart failure development could be explained by proinflammatory cytokine release, endothelial dysfunction, and sympathetic activation exaggerated fluid shifts leading to volume overload [44]. Similarly, lung inflammation as a direct result of influenza infection and immune responses to the virus can interrupt gas exchange in the alveoli, leading to exacerbation of chronic respiratory diseases, and development of acute respiratory distress and increased risk of secondary bacterial pneumonia infections [45]. Other complications of influenza include influenza-associated encephalitis/encephalopathy, Guillain-Barre syndrome, acute kidney injury, myalgias, hepatic, and hematologic complications [46].

While previous studies have not indicated any significant beneficial reduction in overall hospitalization risk following influenza vaccination, results from our study echo those of other studies which have noted significant benefits in reducing exacerbation risks in patients both with and without underlying chronic conditions. In patients with chronic obstructive pulmonary disease (COPD), influenza vaccination has been shown to reduce exacerbations, hospitalizations, and outpatient visits, and decrease all-cause and respiratory mortality [47]. Additionally, influenza vaccination has been associated with a lower risk of major adverse cardiac events (MACE) and cardiovascular-related mortality in high-risk patients, but no impact on myocardial infarction [13,44]. A meta-analysis of randomized clinical trials between 2000 and 2021 showed similar findings, with a 34% reduction in risk of MACE overall in individuals who had been vaccinated against influenza, and a 45% risk reduction in those who had experienced a recent coronary syndrome [48]. Evaluation of high-dose versus standard-dose vaccines has not demonstrated any significant additional benefit of a higher dosage in patients with underlying CVD, with broadly similar rates of hospitalizations and deaths from cardiovascular/pulmonary causes with either vaccine [49]. While our analysis indicates that there could potentially be an incremental reduction in hospitalizations and mortality rates following aQIV compared with QIVe, it is difficult to precisely estimate the potential benefits of the two vaccines. Additionally, one limitation of our study was that we only included older adults with pre-existing comorbidities. While not evaluated in our analysis, it is likely that the influenza vaccines evaluated can also reduce the risk of severe complications in younger patients with high-risk conditions to a similar degree, although this remains an area for future research. Finally, we also did not consider the potential for excess mortality due to patients being turned away from hospital due to lack of available acute beds, nor did we differentiate between ICU and non-ICU hospital admissions. While the latter could have provided further insight into the impact of vaccination on disease severity, reliable published data were not available on acute vs. ICU admissions for individual influenza complications, therefore we did not include this distinction in our model.

One challenge faced when modelling the impact of vaccination on hospitalization and mortality from influenza complications was the uncertainty surrounding the input parameter estimates. In line with this, we decided to adopt a stochastic decision-tree approach, using a range of values for potential vaccine effectiveness, attack rates, and probabilities of events. We believe that this approach allows us to capture uncertainties across seasons, as the effectiveness of vaccines and circulating virus strains varies on a yearly basis. Our objective, therefore, was not to estimate a precise value of the effect of vaccination on reducing hospitalization and mortality rates, but to present a range of possible outcomes across varying influenza epidemiological situations within a given influenza season. The results from our study are in line with published findings on the estimated impact of influenza vaccination in the US in the 2021–2022 influenza season, where 13,614 hospitalizations and 867 deaths were prevented in the ≥65 years age group by vaccination, compared with a no-vaccination scenario [50]. While not providing absolute estimates of the potential risk reduction in severe outcomes of influenza complications, our analysis has shown that vaccination is an important tool in reducing the severity of influenza, and can prevent many severe complications and deaths from influenza-induced exacerbations of existing conditions.

## 5. Conclusions

Our analysis demonstrates the reduction in cardiovascular, respiratory, and other complications in patients ≥65 years of age with influenza infection achieved by immunization with either QIVe or aQIV, with highest impacts from the adjuvanted vaccine. Our model highlights the benefit of vaccination in both the prevention of morbidity and mortality due to influenza as well as the potential to reduce exacerbations of chronic comorbidities such as congestive heart failure and chronic obstructive lung disease. These data add to the understanding of the benefits of seasonal influenza immunization and further encourage primary care clinicians to advocate with their geriatric patients. Furthermore, they may provide perspective for patients to better understand the benefits of influenza immunization for prevention of serious complications of influenza disease, in addition to upper respiratory signs and symptoms.

## Figures and Tables

**Table 1:** Parameters included in the stochastic model.

Variables	Lower Bound	Upper Bound	Distribution	Reference
Incidence	4%	10%	Uniform	[28]
Complications from CVD	17%	50%	Beta	[30]
Complications from respiratory illness	9%	26%	Beta	[30]
Probability of death given CVD	5%	14%	Beta	[31]
Probability of death given respiratory illness	5%	14%	Beta	[31]
Probability of death given all other diseases	5%	14%	Beta	[31]
rVE CVD aQIV	5%	21%	Normal	[23]
VE CVD QIVe	32%	48%	Normal	[32]
rVE respiratory illness aQIV	5%	21%	Normal	[23]
VE respiratory illness QIVe	30%	50%	Normal	[32]
rVE other aQIV	5%	21%	Normal	[23]
VE other QIVe	32%	48%	Normal	[32]

aQIV, adjuvanted quadrivalent influenza vaccine; CVD, cardiovascular disease; QIVe, egg-based quadrivalent influenza vaccine; rVE, relative vaccine effectiveness; VE, vaccine effectiveness. Details of the model distributions are provided in the Supplementary Materials.

## Data Availability

The data presented in this study are available on reasonable request from the corresponding author.

## Supplementary Materials

The following supporting information can be downloaded at: https://www.mdpi.com/article/10.3390/vaccines11101525/s1, Table S1: Estimated median (95% CI) hospitalized cases and deaths in patients ≥65 years of age from respiratory illnesses, cardiovascular disease, and other causes for no vaccination and aQIV vaccination; Table S2: Incremental differences in estimated hospitalization rates and mortality due respiratory, CVD, and other complication of influenza for vaccination of adults ≥65 years with aQIV vs. QIVe.
