# Supplementary material for: The Impact of Adjuvanted Influenza Vaccine on Disease Severity in the US: A Stochastic Model
Source: Vaccines (Basel). 2023 Sep 26;11(10):1525. doi: 10.3390/vaccines11101525 (PMC10610929; doi:10.3390/vaccines11101525)
Supplement: Supplementary file 1 [file vaccines-11-01525-s001.zip › vaccines-2549820-supplementary.pdf]

Mathematical equations used in the stochastic model

1. **Uniform distribution:**

   $x \sim U(a, b)$

   where x is the random variable, a is the lower bound, and b is the upper bound.

2. **Normal distribution:**

   $x \sim N(\mu, \sigma^2)$

   where x is the random variable, μ is the mean, and σ^2 is the variance.

3. **Beta distribution:**

   $x \sim Beta(\alpha, \beta)$

   where x is the random variable, α is the alpha parameter, and β is the beta parameter.

Table S1. Estimated median (95% CI) hospitalized cases and deaths in patients ≥65 years of age from respiratory illnesses, cardiovascular disease, and other causes for no vaccination and aQIV vaccination

Variable	No vaccination	QIVe	aQIV
**Hospitalization**			
Respiratory illness	98,794 (44,347–196,134)	53,455 (22,235–100,237)	50,103 (20,877–94,401)
CVD	190,829 (82,306–378,236)	102,569 (45,951–196,580)	97,120 (43,321–185,025)
Other	299,343 (135,789–533,813)	156,958 (72,016–281,476)	148,189 (68,062–263,408)
**Death**			
Respiratory illness	9,303 (3,193–19,996)	4,815 (1,676–10,494)	4,549 (1,583–9,849)
CVD	17,349 (6,379–40,583)	9,045 (3,279–21,284)	8,543 (3,119–20,062)
Other	26,235 (10,051–56,806)	13,718 (5,277–29,702)	12,840 (4,986–27,807)

aQIV, adjuvanted quadrivalent influenza vaccine; CI, confidence interval; QIVe, egg-based quadrivalent influenza vaccine

Table S2. Incremental differences in estimated hospitalization rates and mortality due respiratory, CVD, and other complication of influenza for vaccination of adults ≥65 years with aQIV vs QIVe

Variable	Median	Lower bound 95% CI	Upper bound 95% CI
**Hospitalization**			
Respiratory illness	2,981	1,071	6,301
CVD	5,747	2,204	12,248
Other	8,768	3,583	18,388
**Death**			
Respiratory illness	272	85	655
CVD	514	172	1,267
Other	765	271	1,944

aQIV, adjuvanted quadrivalent influenza vaccine; CI, confidence interval; QIVe, egg-based quadrivalent influenza vaccine
